# Comparison of Static and Articulating Spacers After Periprosthetic Joint Infection

**DOI:** 10.5435/JAAOSGlobal-D-22-00284

**Published:** 2023-02-06

**Authors:** Hunter S. Warwick, Timothy L. Tan, Lucas Weiser, David N. Shau, Jeffrey J. Barry, Erik N. Hansen

**Affiliations:** From the Department of Orthopaedic Surgery, University of California at San Francisco, San Francisco, CA.

## Abstract

There is no consensus on whether articulating or static spacers are superior during two-stage exchange arthroplasty for periprosthetic joint infection. We aimed to compare surgical time, need for extensile exposure, surgical costs, and treatment success for articulating and static spacers.

**Methods:**

This was a retrospective review of 229 periprosthetic joint infections treated with two-stage exchange with a minimum of one-year follow-up. For articulating and static spacers, we compared the need for extensile exposure during reimplantation and treatment failure based on an updated definition. Surgical time and costs at both stages were also compared. Subgroup analysis was performed for total knee and hip arthroplasties.

**Results:**

There was no difference in the surgical time for spacer insertion; however, articulating spacers demonstrated reduced surgical time during reimplantation (181 vs. 234 minutes, *P* < 0.001). In multivariate analysis, there was no difference in extensile exposures (odds ratio 2.20, *P* = 0.081), but treatment failure was more likely for static spacers (odds ratio 2.17, *P* = 0.009). Overall surgical costs for two-stage exchange were similar between groups (23,782 vs. 23,766, *P* = 0.495).

**Conclusion:**

Articulating spacers demonstrated shorter surgical times and a trend toward decreased extensile exposures during reimplantation. They also had higher treatment success rates and similar surgical costs for overall two-stage exchange.

Periprosthetic joint infection (PJI) remains one of the most devastating complications after total joint arthroplasty.^1,2^ The preferred treatment of chronic PJI in the United States is two-stage exchange arthroplasty.^3^ This involves removal of all components and insertion of an antibiotic-impregnated spacer, followed by an interim period of antibiotics and reimplantation of components at a later date. The reported success rate of two-stage exchange varies, ranging from 65% to 100% depending on inclusion criteria and definition of treatment success.^2,4,5,6,7^ Recent studies suggest that we may be underestimating the attrition, morbidity, and failure rate after two-stage exchange.^1,2,6,8^ New recommendations from the Musculoskeletal Infection Society (MSIS) have created a more encompassing definition of failure.^9^

There are two types of spacers traditionally used, articulating (dynamic) and static. Many types of articulating spacers are available, including handmade or prefabricated all-cement spacers and low-friction spacers containing metal and/or polyethylene. Neither static nor dynamic spacers have been shown to be clearly superior. A recent randomized study by Nahhas et al. found that articulating knee spacers can reduce length of stay, improve final range of motion, and result in better function compared with static spacers. There was no difference in the surgical times between the two spacers, but results suggested there may be increased need for an extensile exposure in patients with static spacers.^10^ Although articulating spacers in knee arthroplasty might achieve increased range of motion and function, there are concerns about their use in cases of instability and bone loss, and multiple studies have found no difference in reinfection rate between static and articulating spacers in the knee.^4,11^ Furthermore, articulating spacers are generally thought to be more costly than static spacers, but costs for the two spacer types have not been examined in detail.^10,12^

Because articulating spacers allow for increased range of motion and soft-tissue pliability, they may be expected to result in easier exposure at the time of reimplantation, which could lead to shorter surgical times and a decreased need for extensile exposure. However, prior randomized studies are likely underpowered to detect differences in these outcomes.^10^ Recent literature suggests reporting outcomes for hip and knee PJIs together with a minimum of 1-year follow-up is reliable and allows for greater sample size and power for evaluation of infrequent outcomes.^13^ Furthermore, the existing body of evidence on static versus articulating spacers uses definitions of treatment success that likely underestimate the rate of failure in two-stage exchange for PJI.^14^ The purpose of the study was to compare static and articulating spacers in surgical time, need for extensile exposure, surgical costs, and treatment success using a broader definition from the MSIS.^9^

## Methods

This was a retrospective study at a single institution for all patients who underwent two-stage exchange arthroplasty from 2004 to 2020 for PJI based on the 2011 definition created by the MSIS.^15^ Patients with a mega-prosthesis or a spacer placed at an outside institution were excluded. All patients were required to have a minimum of one-year follow-up after initial spacer placement. After exclusion of 31 cases, the final cohort included a total of 229 PJIs. This cohort comprised 61 primary total hip arthroplasties (THAs), 91 primary total knee arthroplasties (TKAs), 34 revision THAs, and 43 revision TKAs.

The decision to use an articulating or static spacer was based on surgeon discretion. However, absolute indications for a static spacer included gross instability, lack of ligamentous integrity, and presence of critical soft-tissue defects or recurrent wound healing issues. Articulating spacers were either fashioned by hand, made from preformed molds, or included prosthetic components comprising metal and/or polyethylene. All spacers contained antibiotic-loaded cement.

### Surgical Technique

A thorough synovectomy and débridement was performed, followed by irrigation with 9 L of fluid. Use of additional antimicrobial irrigation solutions was used at surgeon discretion, followed by placement of an antibiotic spacer. Antibiotic type and dose used in spacers was decided by the treating surgeon. When required, extensile exposure, such as a quadriceps snip, tibial tubercle osteotomy, or extended trochanteric osteotomy, was used to facilitate exposure or extraction of components or spacer. A total of 6 to 8 weeks of systemic antibiotics was administered based on culture results and recommendations from an infectious disease consultant. In addition, serum erythrocyte sedimentation rate and C-reactive protein were trended. An antibiotic holiday of 4 to 6 weeks was routinely used before reimplantation, during which time clinical symptoms were monitored. Repeat aspiration during this period was the decision of the treating surgeon because there was no institutional protocol to determine timing of reimplantation. Repeat débridement was performed at time of reimplantation and revision components used. Extensile exposures were used as needed.

### Outcome Variables

Retrospective chart review was performed to obtain surgical details, including surgical times, need for extensile exposures at reimplantation, and type of spacer used. Details on implant and supply costs in USD for both stages were also collected. Dates and clinical course of any subsequent surgeries after initial spacer placement, need for amputation, mortality rates, and organism information from cultures were also collected. Antibiotic-resistant organisms were considered vancomycin-resistant *Enterococcus* and methicillin-resistant *Staphylococcus aureus*.

An electronic query of the medical record was also performed to extract details on comorbidities (diabetes mellitus, hypothyroidism, renal failure, liver disease, anemia, rheumatologic disease, tobacco use, alcohol abuse, drug abuse, psychotic disease, depression, and host grade), age, body mass index, and sex. Patients were classified as either A, B, or C hosts according to the McPherson classification for systemic host grade.^16^

### Primary and Secondary Outcomes

The primary outcomes included surgical time at both the initial spacer insertion and reimplantation, need for an extensile exposure at reimplantation (osteotomy or quadriceps snip), surgical implant and supply costs for both stages, and treatment success for infection. Treatment success was based on the 2019 MSIS definition, where Tier 1 or 2 are defined as a success while Tier 3 or 4 represent failure, which includes patients who were not reimplanted, died, or underwent unplanned reoperations (Table 1).^9^ Surgical time was defined as the time from incision to skin closure. Only new extensile exposures at the time of reimplantation were considered (i.e. reopening of extensile exposures used during stage 1 were not included). Secondary outcomes included mortality and failure to undergo reimplantation.

**Table 1:** Treatment Success Definition

Treatment Success Definition		
Success	Tier 1	Infection control with no continued antibiotic therapy
	Tier 2	Infection control with the patient on suppressive antibiotic therapy
Failure	Tier 3	Need for revision surgery and/or revision and/or spacer retention (assigned to subgroups A, B, C, D, E, and F based on the type of revision surgery)
	A	Aseptic revision at >1 yr from initiation of PJI treatment
	B	Septic revision (including débridement, antibiotics, and implant retention [DAIR]) at >1 yr from initiation of PJI treatment (excluding amputation, resection arthroplasty, and arthrodesis)
	C	Aseptic revision at ≤1 yr from initiation of PJI treatment
	D	Septic revision (including DAIR) at ≤1 yr from initiation of PJI treatment (excluding amputation, resection arthroplasty, and arthrodesis)
	E	Amputation, resection arthroplasty, or arthrodesis
	F	Retained spacer
	Tier 4	Death (assigned to subgroups A or B)
	A	Death <1 yr from initiation of PJI treatment
	B	Death >1 yr from initiation of PJI treatment

PJI = periprosthetic joint infection

### Statistical Analysis

Statistical analysis was performed with SPSS (version 21.0, IBM Corp). Continuous variables were evaluated using Student *t* test or Mann-Whitney *U* test as appropriate. Categorical variables were assessed using a Fisher exact test or Chi-square test. Odds ratios were also calculated.

Univariate analyses were performed to compare demographic and other perioperative variables. A multivariate logistic regression model was used to determine risk factors for treatment failure and need for extensile exposure at reimplantation. The logistic regression included demographic variables, baseline characteristics that differed between static and articulating spacer groups, and variables below a *P*-value threshold of 0.15 in univariate analysis. Two multivariate logistic regression models were performed, with one containing relevant individual comorbidities and one using host grade. Subgroup analysis was performed by joint, for primaries only, and for a cohort that excluded patients with a contraindication to a dynamic spacer, which included the criteria mentioned above. An alpha of 0.05 was used to determine statistical significance.

## Results

**Figure 1:**
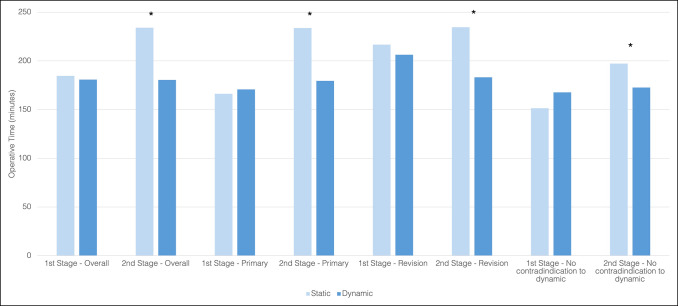
Operating room time for static and dynamic spacers for the overall cohort, infected primaries, infected revisions, and patients with no contraindication to dynamic spacer (* denotes statistical significance at *P* < 0.05).

During the time of initial spacer insertion, a static spacer was used in 43% (n = 99) and an articulating spacer in 57% (n = 130) of patients. For knees, a static spacer was used in 54% (n = 72) of cases and a dynamic spacer was placed in 46% (n = 62) of cases, 47% (n = 29) of which contained metal components. For hips, a static spacer was used in 28% (n = 27) of cases and a dynamic spacer was used in 72% (n = 68) of cases, 87% (n = 59) of which contained metal components. Static spacers were more often used in knees and in patients with diabetes, hypothyroidism, higher American Society of Anesthesiology score, higher host grade, and polymicrobial infections (Table 2). At the time of spacer insertion, there was no difference in the surgical time between static and dynamic spacers for the entire cohort, after infected primaries only, or in patients with no contraindication to a dynamic spacer. However, surgical time at the time of reimplantation was shorter in patients with articulating spacers compared with static spacers for all cases (180.5 ± 47.5 minutes vs. 234.2 ± 65.7 minutes, *P≤*0.001), infected primaries only (179.5 ± 47.9 minutes vs. 233.9 ± 10.1 minutes, *P* < 0.001), and those without a contraindication to a dynamic spacer (172.7 ± 38.0 vs. 197.3 ± 48.5, *P* = 0.008) (Table 3). Revision PJIs also demonstrated similar surgical times for dynamic and static spacers during stage 1 (206.3 ± 78.3 minutes vs. 216.8 ± 96.9 minutes *P* = 0.632) but decreased reimplantation time with dynamic spacers (183.3 ± 47.2 minutes vs. 234.7 ± 66.7 minutes, *P* = 0.003) (Figure 1).

**Table 2:** Demographics and Characteristics by Spacer Type

Demographics	Articulating (n = 130)	Static (n = 99)	*P* value
Knee	47.7% (62/130)	72.7% (72/99)	**<0.001**
Infection of primary	70.8% (92/130)	60.6% (60/99)	0.107
Male	46.9% (61/130)	46.5% (46/99)	0.945
Age	64.2 ± 11.1	63.0 ± 10.1	0.431
BMI	29.6 ± 6.2	30.5 ± 9.1	0.356
DM	12.7% (16/130)	24.3% (24/99)	**0.013**
Hypothyroid	13.1% (17/130)	4.0% (4/99)	**0.020**
Renal failure	15.4% (20/130)	22.2% (22/99)	0.185
Liver disease	10.8% (14/130)	14.1% (14/99)	0.440
AIDS	0.0% (0/130)	1.0% (1/99)	0.251
Anemia	30.0% (39/180)	39.4% (79/180)	0.700
Rheumatological disease	5.4% (7/130)	7.1% (7/99)	0.598
Malnutrition	0.8% (1/130)	1.0% (1/99)	0.846
Alcohol abuse	6.2% (8/130)	6.1% (6/99)	0.977
Drug abuse	7.7% (10/130)	11.1% (11/99)	0.375
Tobacco use	7.7% (10/130)	12.1% (12/99)	0.260
Psychosis	1.5% (2/130)	3.0% (3/99)	0.444
Depression	30.8% (40/130)	27.3% (27/99)	0.565
ASA score	2.5 ± 0.6	2.7 ± 0.6	**0.021**
Host grade			
A	53.1% (69/130)	34.3% (34/99)	**0.005**
B	40.0% (52/130)	58.6% (58/99)	**0.005**
C	6.9% (9/130)	7.1% (7/99)	0.968
Organism			
*Staphylococcus aureus*	28.5% (37/130)	28.3% (28/99)	0.976
Resistant organisms	22.3% (29/130)	33.3% (33/99)	0.063
Polymicrobial	7.7% (10/130)	18.2% (18/99)	**0.016**

Bold entries denote P values below a threshold of 0.05.

BMI = body mass index, AIDS = acquired immunodeficiency syndrome, ASA = American Society of Anesthesiology

**Table 3:** Outcomes by Spacer Type

	Static	Articulating	Odds Ratio	*P*
Surgical time (min)—1st stage			—	
Overall	184.6 ± 73.0	180.9 ± 59.3	—	0.694
Knee	160.5 ± 57.7	176.5 ± 70.2	—	0.180
Hip	198.6 ± 55.3	208.6 ± 77.4	—	0.521
Primaries	166.3 ± 47.0	170.8 ± 46.6	—	0.570
No contraindication to dynamic	151.4 ± 41.7	167.8 ± 43.7	—	0.215
Surgical time (min)—2nd stage			—	
Overall	234.2 ± 65.7	180.5 ± 47.5	—	**<0.001**
Knee	233.4 ± 66.4	179.1 ± 51.8	—	**<0.001**
Hip	237.4 ± 65.2	182.0 ± 43.4	—	**0.001**
Primaries	233.9 ± 10.1	179.5 ± 47.9	—	**<0.001**
No contraindication to dynamic	197.3 ± 48.5	172.7 ± 38.0	—	**0.008**
Treatment failure				
Overall	58.6% (58/99)	36.2% (47/130)	2.50 (1.46-4.27)	**0.001**
Knee	55.6% (40/72)	35.5% (22/62)	2.27 (1.13-4.57)	**0.024**
Hip	66.7% (18/27)	36.8% (25/68)	3.44 (1.34-8.81)	**0.012**
Primaries	56.7% (34/60)	34.8% (32/92)	2.45 (1.26-4.78)	**0.008**
No contraindication to dynamic	60.0% (27/45)	35.3% (31/88)	2.76 (1.32-5.58)	**0.006**
Extensile exposure—2nd stage				
Overall	26.5% (18/68)	13.2% (14/106)	2.37 (1.09-5.15)	**0.028**
Knee	27.8% (15/54)	9.1% (5/55)	3.85 (1.29-11.50)	**0.012**
Hip	21.4% (3/14)	17.6% (9/51)	1.27 (0.29-5.51)	0.747
Primaries	19.5% (8/41)	9.1% (7/77)	2.42 (0.81-7.25)	0.106
No contraindication to dynamic	6.7% (3/45)	5.7% (5/88)	1.19 (0.27-5.20)	0.821
No reimplantation				
Overall	27.3% (27/99)	10.8% (14/130)	3.11 (1.53-6.32)	**0.001**
Knee	20.8% (15/72)	3.2% (2/62)	7.90 (1.73-36.07)	**0.003**
Hip	44.4% (12/27)	17.6% (12/68)	3.73 (1.40–9.97)	**0.010**
Primaries	25.0% (15/60)	10.9% (10/92)	2.73 (1.14-6.58)	**0.022**
No contraindication to dynamic	33.3% (15/45)	11.4% (10/88)	3.90 (1.58-9.63)	**0.002**
Mortality				
Overall	6.1% (6/99)	3.1% (4/130)	2.03 (0.558-7.41)	0.274
Knee	6.9% (5/72)	3.2% (2/62)	2.24 (0.42-11.97)	0.450
Hip	3.7% (1/27)	2.9% (2/68)	1.27 (0.11-14.6)	0.848
Primaries	5.0% (3/60)	3.3% (3/92)	1.56 (0.31-8.01)	0.681
No contraindication to dynamic	4.4% (2/45)	3.4% (3/88)	1.32 (0.21-8.19)	0.766

Bold entries denote P values below a threshold of 0.05.

A total of 15 patients had undergone extensile exposure during stage 1. In the static group, 6.6% of patients (7/99) had a previous extensile exposure, compared with 7% of patients (8/130) in the dynamic group (*P* = 0.795). The rates of extensile exposure at the time of reimplantation were 26.5% (18/68) and 13.2% (14/106) for static and articulating spacers, respectively (Table 3). When controlling for potential confounders in multivariate analysis, static spacers were not significantly associated with increased odds of requiring extensile exposure (OR 2.20, *P* = 0.081). Infected primary arthroplasty was associated with a decreased risk of extensile exposure compared with infected revision (OR 0.320, *P* = 0.008) (Table 4).

**Table 4:** Multivariate Analysis for Extensile Exposure During Reimplantation

	Odds Ratio (95% CI)	*P* value
*Host grade*		
Primary	0.320 (0.138-0.741)	**0.008**
Knee involvement	0.810 (0.336-1.952)	0.638
Male	1.752 (0.760-4.042)	0.188
Age>80	1.307 (0.227-7.523)	0.764
Host grade B or C	1.935 (0.799-4.686)	0.144
Static spacer	2.200 (0.908-5.328)	0.081
Polymicrobial	0.335 (0.077-1.460)	0.145
*Individual comorbidities*		
Primary	0.304 (0.124-0.748)	**0.010**
Knee involvement	0.759 (0.305-1.894)	0.555
Male	1.495 (0.612-3.651)	0.377
Age>80	2.192 (0.369-13.02)	0.388
Diabetes mellitus	1.794 (0.633-5.086)	0.272
Hypothyroidism	0.382 (0.064-2.281)	0.291
Liver disease	2.863 (0.862-9.502)	0.086
Anemia	0.719 (0.269-1.922)	0.511
Drug abuse	2.334 (0.614-8.869)	0.213
Static spacer	2.245 (0.902-5.592)	0.082
Polymicrobial	0.373 (0.080-1.735)	0.209

Bold entries denote P values below a threshold of 0.05.

The overall treatment failure rate for the cohort was 45.9% (105/229). The failure rate was 58.6% (58/99) for the static group compared with 36.2% (47/130) for the dynamic group (Table 3). In the static group, 46.6% (27/58) of failures were from no reimplantation, 32.8% (19/58) from the need for repeat irrigation and débridement or spacer exchange for persistent infection, 10.3% (6/58) due to mortality, 6.7% (5/58) due to revision surgery for a noninfectious reason, and 1.7% (1/58) due to amputation. In the dynamic group, 48.9% (23/47) of failures were from repeat irrigation and débridement or spacer exchange, 29.8% (14/47) from no reimplantation, 10.6% (5/47) due to noninfectious revision surgery, 8.5% (4/47) due to mortality, and 2.1% (1/47) due to amputation. Although static spacers demonstrated an increased risk of failure in the multivariate analysis when using host grade (OR 2.17, *P* = 0.009), there was not an increased risk when using individual comorbidities (OR 1.80, *P* = 0.060) (Table 5).

**Table 5:** Multivariate Analysis for Treatment Failure

	Odds Ratio (95% CI)	*P* value
*Host grade*		
Primary	0.859 (0.478-1.545)	0.612
Knee involvement	0.849 (0.477-1.513)	0.579
Male	0.756 (0.434-1.318)	0.324
Age>80	0.981 (0.315-3.057)	0.973
Host grade B or C	1.941 (1.100-3.423)	**0.022**
Static spacer	2.167 (1.209-3.885)	**0.009**
Antibiotic resistant	1.454 (0.771-2.744)	0.248
Polymicrobial	1.504 (0.648-3.490)	0.342
*Individual comorbidities*		
Primary	0.830 (0.448-1.540)	0.556
Knee involvement	1.003 (0.548-1.836)	0.993
Male	0.625 (0.346-1.127)	0.118
Age>80	1.296 (0.391-4.295)	0.672
Diabetes mellitus	1.394 (0.646-3.007)	0.397
Hypothyroidism	0.369 (0.118-1.151)	0.086
Renal failure	1.940 (0.894-4.209)	0.094
Alcohol abuse	4.282 (1.126-16.29)	**0.033**
Static spacer	1.800 (0.976-3.319)	0.060
Antibiotic resistant	1.469 (0.756-2.855)	0.256
Polymicrobial	1.977 (0.812-4.814)	0.133

Bold entries denote P values below a threshold of 0.05.

When stratified by joint involvement, both hips and knees demonstrated reduced surgical times at reimplantation, lower treatment failure rates, and higher reimplantation rates with articulating spacers. However, extensile exposures at reimplantation were increased for static spacers in knees but not hips. In subgroup analysis of patients without a contraindication to a dynamic spacer, dynamic spacers were associated with a lower rate of failure but not extensile exposures (Table 3).

During stage 1, implant and supply costs were greater for dynamic spacers ($6,682 ± 5,320 vs. $3,153 ± 4,151, *P* < 0.001). During stage 2, costs were higher for static spacers ($22,229 ± 7,180 vs. $17,493 ± 5,892, *P* < 0.001). Combined costs for both stages were similar for static and dynamic spacers. These results were similar when stratified by joint involvement (Table 6).

**Table 6:** Surgical Costs by Spacer Type

	Static	Articulating	*P* value
Surgical cost (USD)—1st stage			
Overall	3,153 ± 4,151	6,682 ± 5,320	**<0.001**
Knee	2,867 ± 3,052	5,521 ± 5,572	**<0.001**
Hip	3,949 ± 6,307	7,739 ± 4,891	**<0.001**
Surgical cost (USD)—2nd stage			
Overall	22,229 ± 7,180	17,493 ± 5,892	**<0.001**
Knee	22,201 ± 6,201	20,480 ± 6,006	**0.048**
Hip	22,337 ± 10,478	14,020 ± 3,314	**<0.001**
Total surgical cost (USD)			
Overall	23,782 ± 6,615	23,766 ± 8,530	0.495
Knee	23,979 ± 6,351	26,099 ± 9,925	0.491
Hip	22,977 ± 7,899	20,834 ± 5,130	0.488

Bold entries denote P values below a threshold of 0.05.

There was no difference in the mortality rate between static spacers and articulating spacers (Table 3). However, patients with a static spacer were less likely to be reimplanted (10.8% vs. 27.3%, *P* = 0.001).

## Discussion

There is no consensus on whether articulating or static spacers should be used during two-stage exchange arthroplasty. We aimed to evaluate whether the use of articulating spacers influences variables related to the reimplantation procedure. We found that dynamic spacers demonstrated lower surgical times and a trend toward fewer extensile exposures at reimplantation. Furthermore, articulating spacers had higher rates of reimplantation and treatment success with similar costs for overall two-stage exchange.

Many studies have compared range of motion, functional outcomes, and treatment success between articulating and static spacers in revision knee arthroplasty.^10–12,17–19^ Multiple systematic reviews have demonstrated greater range of motion after reimplantation for articulating spacers.^4,11^ In a prospective randomized control trial of 68 knee PJIs, Nahhas et al.^10^ found that patients with an articulating spacer demonstrated higher Knee Society Scores, range of motion, and decreased length of stay. However, most studies demonstrate no difference in the reinfection rate between spacer type.^4,11^ By contrast, our study found a higher treatment failure rate with static spacers. This finding has multiple contributing explanations. First, patients with indicators of worse infection, such as greater bone loss and more extensive soft-tissue damage, are more likely to receive a static spacer. In addition, we used a different definition of failure in this study, which included reinfection, patients who never underwent reimplantation, noninfectious reoperations, and patient mortality. This new MSIS definition has been shown to result in detection of fewer instances of successful treatment than prior methods.^14^ Indeed, the failure rate in our study was higher than reported elsewhere, likely a result of the broader definition used as well as the inclusion of infected revision arthroplasties, which comprised a third of our overall cohort.

Our study suggests that articulating spacers may facilitate easier reimplantation. We found a reduction in surgical time at reimplantation after the placement of articulating spacers. Few other studies have reported on surgical time. In the previously mentioned randomized control trial by Nahhas et al., they found no difference in the surgical time between articulating and static spacers in TKA both at stage 1 (132 vs. 142 minutes) and at stage 2 (143 vs. 145 minutes). However, although not significant, they found that static spacers were associated with a greater need for extensile exposure at reimplantation (16.7% vs. 4.0%, *P* = 0.189).^10^ In a systematic review of 622 articulating and 203 static knee spacers, Guild et al.^12^ found that an extensile exposure or rotational flap was needed in 24% of patients with dynamic spacers compared with 35% of those with static spacers (*P* < 0.0011). We found a trend toward increased risk of extensile exposure with static spacers, but this association was not statistically significant. Extensile exposures and surgical time are both surrogate measures for difficulty of reimplantation. Differences in surgical time at stage 2 for static and dynamic spacers likely represent extensile exposures as well as procedural complexity and soft-tissue releases not quantified in surgical reports; therefore, surgical time may reflect the difficulty of the exposure and operation more accurately than extensile exposures alone. Infected revision arthroplasty was the only variable associated with higher utilization of extensile exposure, likely the result of substantial scar tissue and soft-tissue contracture often present in revision cases.

When stratified by joint, the differences between static and articulating spacers were similar for hips and knees, with the exception of extensile exposures during reimplantation. Rates of extensile exposure were higher for static spacers in knees but not hips. This is likely because static spacers of the hip still allow for a certain degree of joint motion, unlike static spacers of the knee. Therefore, one might expect similar rates of extensile exposures for static and articulating hip spacers.

Articulating spacers are commonly cited as incurring higher surgical costs than static spacers, although data to support this claim are lacking.^10,12^ We found that articulating spacers had higher implant and supply costs during stage 1 but lower costs for stage 2. Increased surgical costs for articulating spacers during stage 1 is likely from the use of preformed molds or metal and polyethylene components in many articulating constructs. Higher costs for static spacers during stage 2 may result from the use of more complex components often required to account for greater degrees of bone loss and soft tissue incompetence frequently present in patients with static spacers.^20^ The differences in surgical costs between static and dynamic spacers in stages 1 and 2 appear to balance out overall because total implant and supply costs for two-stage exchange were similar between groups.

There are several limitations that should be considered when interpreting the results of this study. First, the study was retrospective in nature and not a prospective randomized control study. However, the retrospective nature does allow for an increased sample size and power to assess for infrequent outcomes, such as treatment failure and need for extensile exposure. Second, there is a degree of selection bias, as static spacers are frequently used in the setting of severe bone loss, poor host grade, tenuous soft-tissue envelope, and concerns about instability. We accounted for whether the spacer was performed after a primary or revision total joint arthroplasty and performed subgroup analysis for patients without a contraindication to a dynamic spacer, but the possibility of selection bias is nevertheless present. Moreover, patients with static spacers often had increased comorbidities, which may influence results despite our best efforts to control for host differences with multivariate analysis. Third, many of the surgical techniques and decisions on component use, including whether an articulating or static spacer was used, were based on surgeon discretion because there is no standard of care established. Finally, it is difficult to compare the treatment failure rate of our study with prior studies because we used a recent definition of treatment failure that is more inclusive.

In conclusion, there was no difference in surgical time between static and dynamic spacer insertion; however, during reimplantation, dynamic spacers demonstrated a shorter surgical time and a trend toward decreased need for extensile exposure. Further, the treatment failure rate was lower for articulating spacers using a more encompassing definition for this measure, and overall implant and supply costs for two-stage exchange with static and dynamic spacers were similar. These benefits may warrant the use of an articulating spacer when placement is not otherwise contraindicated.

## References

[1] Zmistowski B Karam JA Durinka JB Casper DS Parvizi J: Periprosthetic joint infection increases the risk of one-year mortality. J Bone Joint Surg 2013;95:2177-2184. 24352771 10.2106/JBJS.L.00789

[2] Gomez MM Tan TL Manrique J Deirmengian GK Parvizi J: The fate of spacers in the treatment of periprosthetic joint infection. J Bone Joint Surg 2015;97:1495-1502. 26378265 10.2106/JBJS.N.00958

[3] Abdel MP Barreira P Battenberg A : Hip and knee section, treatment, two-stage exchange spacer-related: Proceedings of international consensus on orthopedic infections. J Arthroplasty 2019;34:S427-S438. 30348562 10.1016/j.arth.2018.09.027

[4] Voleti PB Baldwin KD Lee G-C: Use of static or articulating spacers for infection following total knee arthroplasty: A systematic literature review. J Bone Joint Surg 2013;95:1594-1599. 24005200 10.2106/JBJS.L.01461

[5] Berend KR Lombardi AV Morris MJ Bergeson AG Adams JB Sneller MA: Two-stage treatment of hip periprosthetic joint infection is associated with a high rate of infection control but high mortality. Clin Orthop Relat Res 2013;471:510-518. 22983683 10.1007/s11999-012-2595-x PMC3549176

[6] Wang Q Goswami K Kuo F-C Xu C Tan TL Parvizi J: Two-stage exchange arthroplasty for periprosthetic joint infection: The rate and reason for the attrition after the first stage. J Arthroplasty 2019;34:2749-2756. 31285090 10.1016/j.arth.2019.06.021

[7] Wolf M Clar H Friesenbichler J : Prosthetic joint infection following total hip replacement: Results of one-stage versus two-stage exchange. Int Orthopaedics 2014;38:1363-1368. 10.1007/s00264-014-2309-y PMC4071490 24638215

[8] Corona PS Vicente M Carrera L Rodríguez-Pardo D Corró S: Current actual success rate of the two-stage exchange arthroplasty strategy in chronic hip and knee periprosthetic joint infection. Bone Joint J 2020;102-B:1682-1688. 33249903 10.1302/0301-620X.102B12.BJJ-2020-0792.R1

[9] Fillingham YA Della Valle CJ Suleiman LI : Definition of successful infection management and guidelines for reporting of outcomes after surgical treatment of periprosthetic joint infection: From the workgroup of the musculoskeletal infection society (MSIS). J Bone Joint Surg 2019;101:e69. 31318814 10.2106/JBJS.19.00062

[10] Nahhas CR Chalmers PN Parvizi J : A randomized trial of static and articulating spacers for the treatment of infection following total knee arthroplasty. J Bone Joint Surg 2020;102:778-787. 32379118 10.2106/JBJS.19.00915

[11] Pivec R Naziri Q Issa K Banerjee S Mont MA: Systematic review comparing static and articulating spacers used for revision of infected total knee arthroplasty. J Arthroplasty 2014;29:553-557. 24011676 10.1016/j.arth.2013.07.041

[12] Guild GN Wu B Scuderi GR: Articulating vs. Static antibiotic impregnated spacers in revision total knee arthroplasty for sepsis. A systematic review. J Arthroplasty 2014;29:558-563. 24268975 10.1016/j.arth.2013.08.013

[13] Xu C Tan TL Li WT Goswami K Parvizi J: Reporting outcomes of treatment for periprosthetic joint infection of the knee and hip together with a minimum 1-year follow-up is reliable. J Arthroplasty 2020;35:1906-1911. 32229149 10.1016/j.arth.2020.02.017

[14] Borsinger TM Pierce DA Hanson TM Werth PM Orem AR Moschetti WE: Is the proportion of patients with “successful” outcomes after two-stage revision for prosthetic joint infection different when applying the musculoskeletal infection society outcome reporting tool compared with the delphi-based consensus criteria?. Clin Orthop Relat Res 2021;479:1589-1597. 33543876 10.1097/CORR.0000000000001654 PMC8208431

[15] Parvizi J Zmistowski B Berbari EF : New definition for periprosthetic joint infection: From the workgroup of the musculoskeletal infection society. Clin Orthop Relat Res 2011;469:2992-2994. 21938532 10.1007/s11999-011-2102-9 PMC3183178

[16] McPherson EJ Woodson C Holtom P Roidis N Shufelt C Patzakis M: Periprosthetic total hip infection: Outcomes using a staging system. Clin Orthopaedics Relat Res 2002;403:8-15. 12360001

[17] Ding H Yao J Chang W Liu F: Comparison of the efficacy of static versus articular spacers in two-stage revision surgery for the treatment of infection following total knee arthroplasty: A meta-analysis. J Orthop Surg Res 2017;12:151. 29041970 10.1186/s13018-017-0644-6 PMC5646138

[18] Freeman MG Fehring TK Odum SM Fehring K Griffin WL Mason JB: Functional advantage of articulating versus static spacers in 2-stage revision for total knee arthroplasty infection. J Arthroplasty 2007;22:1116-1121. 18078879 10.1016/j.arth.2007.04.009

[19] Fehring TK Odum S Calton TF Mason JB: Articulating versus static spacers in revision total knee arthroplasty for sepsis. Clin Orthopaedics Relat Res 2000;380:9-16. 10.1097/00003086-200011000-00003 11064968

[20] Calton TF Fehring TK Griffin WL: Bone loss associated with the use of spacer blocks in infected total knee arthroplasty. Clin Orthop Relat Res 1997;345:148-154. 9418632

